# Dynamic Change of Awareness during Meditation Techniques: Neural and Physiological Correlates

**DOI:** 10.3389/fnhum.2012.00131

**Published:** 2012-09-17

**Authors:** Ravinder Jerath, Vernon A. Barnes, David Dillard-Wright, Shivani Jerath, Brittany Hamilton

**Affiliations:** ^1^Department of Obstetrics and Gynecology, Augusta Women's CenterAugusta, GA, USA; ^2^Georgia Prevention Center, Institute of Public and Preventive Health, Georgia Health Sciences UniversityAugusta, GA, USA; ^3^University of South CarolinaAiken, SC, USA; ^4^College of Medicine, American University of AntiguaNew York, NY, USA

## Introduction

Recent findings illustrate how changes in consciousness accommodated by neural correlates and plasticity of the brain advance a model of perceptual change as a function of meditative practice. During the mind-body response neural correlates of changing awareness illustrate how the autonomic nervous system shifts from a sympathetic dominant to a parasympathetic dominant state. Expansion of awareness during the practice of meditation techniques can be linked to the Default Mode Network (DMN), a network of brain regions that is active when the one is not focused on the outside world and the brain is restful yet awake (Chen et al., 2008). A model is presented illustrating the dynamic mind-body response before and after mindfulness meditation, and connections are made with prefrontal cortex activity, the cardiac and respiratory center, the thalamus and amygdala, the DMN and cortical function connectivity. The default status of the DMN changes corresponding to autonomic modulation resulting from meditation practice.

## Modeling Spatial Awareness during the Mind-Body Response

**Figure 1:**
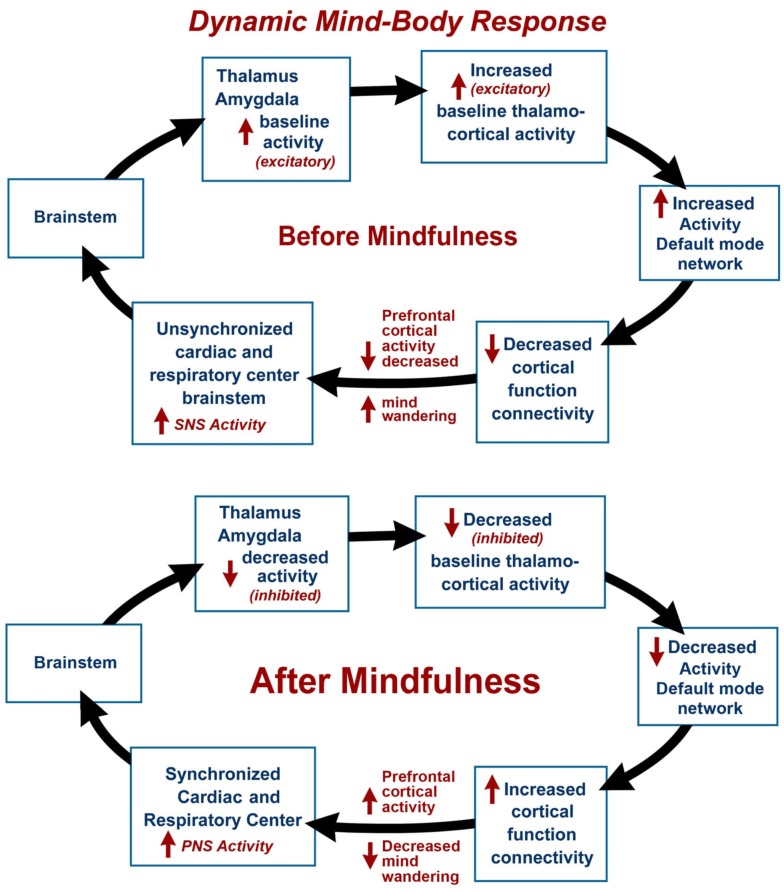
**The dynamic mind-body response is illustrated by a hypothetical psychophysiological condition before mindfulness meditation, with mind wandering and decreased prefrontal cortex activity, leading to unsynchronized cardiac and respiratory centers (elevated sympathetic nervous system activity) and increased activity of the thalamus and amygdala associated with baseline or increased activity of the Default Mode Network (DMN) and decreased cortical function connectivity**. In the final stage and after meditation, there is decreased mind wandering and increased prefrontal cortex activity, leading to synchronized cardiac and respiratory centers (elevated parasympathetic nervous system activity) and decreased activity of the thalamus and amygdala associated with decreased DMN activity and increased cortical function connectivity.

The dynamic mind-body response supports the interrelationship between one's physical health and the state of one's mind. The mind-body response may be illustrated by a hypothetical psychophysiological condition before meditation, with decreased prefrontal cortex activity, with increased mind wandering (Hasenkamp et al., 2012) leading to an unsynchronized cardiac and respiratory center (elevated sympathetic nervous system activity) and increased activity of the thalamus and amygdala (see Figure 1). Increased thalamo-cortical activity is associated with baseline or increased DMN activity and decreased cortical function connectivity. During and after meditation, DMN activity is decreased and there is increased prefrontal cortex activity, leading to a more synchronized cardiac and respiratory center (elevated parasympathetic nervous system activity) and decreased activity of the thalamus and amygdala. This decreased thalamo-cortical activity is associated with decreased DMN activity and increased cortical function connectivity. This model is supported by a number of recent fMRI and other imaging studies.

## fMRI and Other Imaging Findings in Mind-Body Practices

Studies using fMRI suggest that, due to the plasticity of brain, there are dynamic changes in activation of the bilateral temporoparietal junction and the temporal gyri frontal eye field when an individual closes and opens his eyes (Mayer et al., 2004). Endogenous (internal) vs. exogenous (external) facilitation (referring to speed of orienting response) results in widespread cortical activation including the bilateral temporoparietal junction, bilateral superior temporal gyrus, right middle temporal gyrus, right frontal eye field, and left intraparietal sulcus (Mayer et al., 2004). Conversely, exogenous visual space orientation compared to endogenous facilitation results in only a single focus of activation in the left superior temporal gyrus. In our model, the normal activation resulting from endogenous orientation at the start of meditation with eyes closed shifts from the temporal (somatosensory) gyri to the parietal and visual cortex.

fMRI evidence suggests increased blood flow in frontal and parietal lobes after focused meditation (Newberg et al., 2010). A study conducted with mindfulness-based stress reduction on patients with social anxiety disorder reported that during a breath-focused attention task, subjects showed (a) decreased negative emotional experiences, (b) reduced amygdala activity, and (c) increased activity in brain regions implicated in attentional deployment (Goldin and Gross, 2010). Mindfulness meditation has been shown to: (1) decrease pain-related activity of the primary somatosensory cortex, (2) increase activity of the anterior cingulate cortex and anterior insula, and (3) reduce activity of the limbic gating mechanism with the thalamus (amygdala). The findings suggest that mindfulness meditation improves visuo-spatial processing, working memory, and executive functioning (Zeidan et al., 2010). Mindfulness practitioners exhibit lower trait frontal gamma activity, as well as state and trait increases in posterior gamma power, irrespective of practice proficiency suggesting that mindfulness induces neuroplasticity in self-referential and attentional networks (Berkovich-Ohana et al., 2012).

In meditation, cardio-respiratory synchronization leads to a long-lasting neural response as indicated by EEG, fMRI, and other imaging techniques. In an fMRI study where the effects of respiration variations on independent component analysis of DMN were analyzed, it was shown that functional connectivity can be greatly affected by cardiac and respiratory activity (Birn et al., 2008). Regarding coupling between the hemodynamic response and neuronal activity (neurovascular coupling), findings suggest that the hemodynamic response is significantly correlated with neuronal activity. This concept is further illustrated by recent findings of effects of meditation on the DMN.

## The Default Mode Network in Meditation Practice

The DMN is a specific, anatomically defined network of brain regions preferentially active when one is wakeful but resting and not focused on the external environment (Buckner et al., 2008). The DMN is characterized by low frequency 0.1 Hz blood-oxygen-level dependent (BOLD) fMRI signal fluctuations within brain regions (Chen et al., 2008). These fluctuations are hypothesized to result from neuronal activity synchronized within and across brain regions (Birn et al., 2006). Independent component analysis has distinguished waveform activity within the DMN and respiratory induced changes (Birn et al., 2008). It is our hypothesis that respiratory impulses synchronize with the DMN via activity of slowly adapting stretch receptors (SARs) in the lungs. These receptors are activated during inspiration and expiration (Schelegle, 2003). Animal-based studies have shown the origin of rhythmically generated inhibitory impulses by inflation of respiratory alveoli in the lungs, that are detected in the brainstem and other areas of the brain. These neuronal impulses inhibit over-inflation and are involved with the Hering–Breuer reflex (Hering and Breuer, 1868). The DMN average frequency of 0.1 Hz synchronizes activity in widespread regions of the brain and is highly correlated with respiration. Our model suggests that decreased DMN activity during and after mindfulness meditation may lead to increased cortical function connectivity (Figure 1). After meditation, there is a decreased rate of breathing, and decreased activity of the DMN leads to selective inhibition of thalamus and amygdala (sensory relay center and emotional centers respectively) and activation of cortical function connectivity.

Different mind-body techniques may be expected to produce differences in physiological changes as well as different experiences and brain states (Orme-Johnson and Walton, 1998) and these may also be attributed to variations in methodology (Travis et al., 2009). One factor that seems to be common among different types of meditation is respiratory and cardiac synchronization, a feature that underlies increased heart rate variability that is indicative of autonomic modulation. Cardio-respiratory phase synchronization has been shown to be enhanced during meditation, compared with normal relaxation (Wu and Lo, 2010). In Zen and *Kinhin* meditation, the breathing frequency decreases spontaneously even in novices leading to the cardio-respiratory synchronization (Cysarz and Büssing, 2005). The activity of pulmonary SARs is also known to be transmitted to the heart where respiratory sinus arrhythmia is observed during lung inflation. Central and autonomic nervous system interaction has been shown to be impacted by only 5 days of meditation, including lower heart rate and increased heart rate variability (Tang et al., 2009). Cardiorespiratory modulation synchronizes with the DMN and causes increased cortical function connectivity.

The physiological response from the Yogic breathing technique of *pranayama* leads to an autonomic shift from sympathetic to parasympathetic dominance (Jerath et al., 2006), e.g., slower breathing rate (<10 breaths per min) reduces sympathetic and increases parasympathetic activity (Pramanik et al., 2009). The change to a calmer state of mind can be induced by synchronization of hemodynamic changes to slower heart and breathing rates – a higher degree of cardiorespiratory synchronization during meditation (Cysarz and Büssing, 2005). Awareness of the mental transformation leads to a state of increasing calm and outer focus via increased self-awareness through *pranayama* practice (Brown and Gerbarg, 2005).

Variations in brain wave patterns have been observed in different meditation techniques, for example, increased alpha coherence in Transcendental Meditation^®^, higher theta wave activity in Zen meditation and increased gamma activity in mindfulness meditation (Travis and Shear, 2010). Alpha and theta power increase with eyes closed compared to eyes open in normal subjects tested via fMRI scans to monitor the DMN, suggesting a network of spectral EEG activities simultaneously operative at well defined regional fields in the eyes closed state, varying specifically between eyes closed and eyes open states (Chen et al., 2008). The amount of deactivation within the DMN is modulated by the effort required to perform the task.

This feature may help explain why effort in meditation (e.g., concentration) has been shown to decrease activation of the DMN as compared to DMN activation in techniques requiring no effort. The TM technique utilizes automatic self-transcending to allow the mind to settle to a state of quiescence (Alexander et al., 1987). This experience is associated with significantly increased EEG coherence and physiological rest (Travis et al., 2001). Alpha EEG coherence and synchrony, as seen during the TM technique, is associated with neural communication and integration which may be the neurophysiological basis of beneficial physiological and clinical effects of TM (Travis and Shear, 2010). TM produces higher alpha power in the frontal cortex and lower beta and gamma waves in the same frontal areas during practice, and creates greater alpha coherence between the left and right hemispheres of the brain.

Activation of the DMN has been found to be increased during periods of low cognitive load and decreased under greater executive control. Findings reflect differences in specific meditation practices employed, which may also be attributed to methodological differences in measurement of fluctuating cognitive states (Hasenkamp et al., 2012), and meditation studies including TM have shown increased DMN activity during meditation practice (Baron Short et al., 2010; Travis et al., 2010). The practice of TM has been shown to increase functional connectivity of DMN and to activate DMN compared to simple eyes closed rest (Travis et al., 2010). A positron emission tomography study reported that TM practice increases blood flow in the prefrontal cortex, reduces activity in the thalamus and the medial occipital lobe, and reduces hippocampal activity (Newberg et al., 2006). Increased brain wave coherence, commonly seen during TM (Travis et al., 2002), has been reported to “stabilize” in activity outside of meditation. Regular practice engaging DMN over time may induce neuroplastic changes that improve health for the mind and body.

## Conclusion

Recent research illustrates how the neural correlates and plasticity of the brain accommodate changes in consciousness. Neural correlates of expanding awareness during the mind-body response illustrate how the autonomic nervous system shifts from a sympathetic dominant to a parasympathetic dominant state. We have attempted to synthesize the literature in order to advance a model illustrating meditation's dynamic mind-body response, and associations are made with the cardiac and respiratory center, the thalamus and amygdala, the DMN and cortical function connectivity.

Synchronization of the hemodynamic response during meditation was shown to lead to inhibition of the limbic system, and deactivation of DMN leads to an increase in cortical functional connectivity. This article provides evidence to support the mechanism of neurophysiological changes during meditation at the cellular level based on neurovascular coupling, and at the global brain activity level from the autonomic response generated by cardiorespiratory synchronization. Future research will benefit from use of a standardized set of psychophysiological variables and imaging protocols, for comparison of studies different kinds of meditation, that would foster clearer understanding of the relationship between the DMN and experiences during meditation (Travis et al., 2009).
